# Anterior insula stimulation suppresses appetitive behavior while inducing forebrain activation in alcohol-preferring rats

**DOI:** 10.1038/s41398-020-0833-7

**Published:** 2020-05-18

**Authors:** Mia Haaranen, Giulia Scuppa, Stefano Tambalo, Vilja Järvi, Sine M. Bertozzi, Andrea Armirotti, Wolfgang H. Sommer, Angelo Bifone, Petri Hyytiä

**Affiliations:** 1 Department of Pharmacology, Faculty of Medicine, University of Helsinki, Helsinki, Finland; 2 Center for Neuroscience and Cognitive Systems, Istituto Italiano di Tecnologia, Rovereto (TN), Italy; 3 Department of Analytical Chemistry, Fondazione Istituto Italiano di Tecnologia, Genova, Italy; 4 Institute of Psychopharmacology and Department of Addictive Behavior and Addiction Medicine, Central Institute of Mental Health, University of Heidelberg, Medical Faculty Mannheim, Mannheim, Germany; 5 Department of Molecular Biotechnology and Health Sciences, University of Torino, Genova, Italy

**Keywords:** Neuroscience, Pathogenesis

## Abstract

The anterior insular cortex plays a key role in the representation of interoceptive effects of drug and natural rewards and their integration with attention, executive function, and emotions, making it a potential target region for intervention to control appetitive behaviors. Here, we investigated the effects of chemogenetic stimulation or inhibition of the anterior insula on alcohol and sucrose consumption. Excitatory or inhibitory designer receptors (DREADDs) were expressed in the anterior insula of alcohol-preferring rats by means of adenovirus-mediated gene transfer. Rats had access to either alcohol or sucrose solution during intermittent sessions. To characterize the brain network recruited by chemogenetic insula stimulation we measured brain-wide activation patterns using pharmacological magnetic resonance imaging (phMRI) and c-Fos immunohistochemistry. Anterior insula stimulation by the excitatory Gq-DREADDs significantly attenuated both alcohol and sucrose consumption, whereas the inhibitory Gi-DREADDs had no effects. In contrast, anterior insula stimulation failed to alter locomotor activity or deprivation-induced water drinking. phMRI and c-Fos immunohistochemistry revealed downstream activation of the posterior insula and medial prefrontal cortex, as well as of the mediodorsal thalamus and amygdala. Our results show the critical role of the anterior insula in regulating reward-directed behavior and delineate an insula-centered functional network associated with the effects of insula stimulation. From a translational perspective, our data demonstrate the therapeutic potential of circuit-based interventions and suggest that potentiation of insula excitability with neuromodulatory methods, such as repetitive transcranial magnetic stimulation (rTMS), could be useful in the treatment of alcohol use disorders.

## Introduction

The insular cortex (insula) has been implicated in many neuropsychiatric disorders, including addiction. Insula is hypothesized to retrieve the salient interoceptive effects associated with drug and natural rewards and to represent them in the service of goal-directed behavior^1,2^. Smokers with an insula-damaging stroke were more likely to quit smoking compared to patients suffering an insula-sparing stroke, revealing the critical role of the insula in addictive behavior^3^. Similarly, in animal models of addiction, insula lesions or inactivation abolished preference for nicotine- and amphetamine-paired environments in conditioned place preference models^4,5^, and reduced nicotine, alcohol, and cocaine self-administration and seeking^6–8^. In human functional magnetic resonance imaging (MRI) studies, both alcohol- and nicotine-associated cues elicited insula activation^9,10^, suggesting that insula is involved in cue-induced drug craving.

Decreased addictive behavior produced by disruptions of insula function seems to contradict the human neuroimaging data showing that reduced insula volume or reactivity is associated with drug seeking and relapse to drug use following abstinence. For example, lower insula gray matter volume was found in cocaine, heroin, methamphetamine, and cannabis users, as well as alcohol-dependent patients^11–15^. Furthermore, functional neuroimaging suggested that alcohol- and methamphetamine-dependent patients exhibited lower insula activation while performing decision-making and risk evaluation tasks^16,17^. These data can be interpreted as desensitized insula function in drug-addicted individuals.

These seemingly disparate findings of both sensitized and desensitized insula functions in addiction could be understood within a framework in which insula can undergo gain of function, or alternatively, loss of function depending on the stage of addiction and the context^18^. Insula may exhibit enhanced activation during incentive motivational processing, such as cue-induced craving, but have deficits in processing information about the negative consequences of drug taking. This dichotomy is also reflected in the continuing debate whether transcranial magnetic stimulation (TMS) should use stimulation or inhibition for modulation of insula excitability to provide therapeutic efficacy^19^. In addition, development of novel neuromodulatory approaches requires understanding of the functions of the brain networks these methods are targeting. Therefore, we used here a highly integrative, multi-level approach that combined site-specific modulation of insula function and two independent but complementary methods for mapping brain activity following insula interference.

First, we tested the effects of both the anterior insula stimulation and inhibition on alcohol and sucrose consumption with Designer Receptors Exclusively Activated by Designer Drugs (DREADDs)^20^, expressed in the anterior insula, and activated by clozapine-N-oxide (CNO) in selectively bred alcohol-preferring AA rats (Alko Alcohol)^21^. We chose this animal model, because the selection pressure for high alcohol drinking in this rat strain is supposed to enrich the alleles promoting alcohol drinking and may thus partially represent the genetic influence on human alcohol use disorders^22^. We targeted our DREADD modulation at the anterior part of the insula that differs from the posterior insula in cytoarchitecture and functions. The posterior insula is dominated by the granular region with the typical six-layer cortical structure, which becomes thinner in more rostral regions, and disappears in the anterior agranular insula^1^. Functionally, the anterior insula is associated with goal-directed and drug-seeking behavior^6,8^, whereas the posterior section processes aversive sensory stimuli and affective states^23^. Because the consumption of alcohol and natural rewards such as sweet substances are controlled by highly overlapping brain circuits^24,25^ and share common genetic factors^26^, we hypothesized that this commonality may also be reflected in insula functions.

Second, as the insula is a cortical hub with rich connectivity^27^, our second goal was to delineate the insula-centered functional network affected by insula stimulation using a data-driven approach. We accomplished this by pharmacological MRI (phMRI), a functional imaging method that has been applied extensively to probe the activity of specific brain circuitries and neurotransmitter systems in animal models of human brain diseases^28–30^. In addition, we mapped downstream activation of insula stimulation with c-Fos immunohistochemistry. We expected these experiments to elucidate the role of the anterior insula and its connections in alcohol drinking and reward-directed behavior in general, and to guide circuit-based therapeutic manipulations involving the insula to reduce alcohol consumption or craving in alcohol use disorder patients.

## Materials and methods

### Animals

In total, 74 male alcohol-preferring AA (Alko Alcohol) rats (University of Helsinki, Finland) were used for the behavioral experiments. For examining DREADD-induced brain activation, additional 9 rats were used for c-Fos immunohistochemistry and 28 rats (16 with DREADD expression and 12 sham-treated) for phMRI. On arrival, the animals were approximately 10 weeks old with an average weight of 330 g. Throughout the experiments, the rats were weighed once a week. The animals were single housed in transparent, individually ventilated cages (IVCs) containing woodchip bedding, nesting material, a wooden stick, and a plastic tube for enrichment. The rats had ad libitum access to food (SDS, Witham, UK) and water, except during the initial 4 days of forced ethanol drinking when water access was restricted. The animals were maintained at the animal facility of the University of Helsinki (Helsinki, Finland) at a constant temperature of 20 ± 1 °C and a relative humidity of 55 ± 10% with a 12-h light/dark cycle with lights on at 06:00 hours. The experimental procedures were authorized by the project authorization board of the Regional State Administration Agency for Southern Finland and by the Italian Ministry of Health (approval number: 827/2016) and followed the directive 2010/63/EU of the European Parliament and of the Council and the Finnish Act on the Protection of Animals Used for Science or Educational Purposes (497/2013). Except for the alcohol drinking data that consist of two replicated experiments, no other data were replicated.

### Drugs

For the alcohol drinking experiment, 96% ethanol (WWR International, Fontaney-sur-Bois, France) was diluted with tap water for the final concentration of 10% (v/v). CNO (Abcam, Cambridge, UK) was dissolved in saline and administered intraperitoneally at the dose of 10 mg/kg^31^, 60 min before the beginning of the two bottle choice between ethanol or sucrose and water, and 90 min before sacrificing the animals in the c-Fos experiment. For all CNO injections and behavioral testing, the persons conducting the procedures were blind to the group allocations of animals.

For the phMRI experiment, CNO was administered intravenously (i.v.) at a dose of 0.5 mg/kg in 0.5 ml. The i.v. 0.5 mg/kg dose of CNO was chosen, because it did not show any significant phMRI response in rats without DREADDs (*n* = 10) in a pilot experiment (Supplementary Fig. S1). Moreover, the same dose was also used in phMRI experiments with hM3Dq conditional knock-in mice^32^. In addition, pharmacokinetic profiling of the two CNO doses used in this study demonstrated sustained and comparable plasma levels of CNO throughout the imaging and behavioral assessment periods (Supplementary Fig. S2). In agreement with previous data, we also saw conversion of CNO to clozapine that has been implicated in DREADD activation^33^.

### Voluntary alcohol and sucrose drinking

IVCs were equipped with openings for two drinking bottles. During the initial 4-day acquisition phase, rats were presented with two 350 ml bottles equipped with stainless steel spouts and filled with 10% (v/v) ethanol to habituate the animals to the taste of alcohol. After this phase, a two-bottle choice paradigm was introduced, in which rats were given a choice between ethanol and water during 2-h sessions every second day. The fluids were offered in custom-made pipettes with stainless steel spouts, allowing measurement of fluid intake to the nearest 0.1 ml. Each drinking session, the left–right position of the pipettes was changed to prevent side preference development. Between the sessions, rats were presented with two water bottles. The intermittent alcohol drinking was continued for approximately 10 weeks, after which the viral vectors for DREADDs (Gq-DREADD, *n* = 11; Gi-DREADD, *n* = 10) and the control vector for EGFP (*n* = 9) were injected (see below). In all drinking experiments, experimental groups receiving different DREADDs were matched for their baseline consumption during the last week preceding surgery.

Sucrose solutions were offered in similar 2-h two-bottle choice sessions every second day using similar drinking pipettes. Sucrose was first given as a 10% (w/v) solution for four sessions, after which the concentration was decreased to 7.5% and then 5% for five sessions each before the CNO challenge to the three experimental groups (EGFP, *n* = 10; Gq-DREADD, *n* = 10; Gi-DREADD, *n* = 8).

### Deprivation-induced water drinking

Rats expressing either Gq-DREADDS (*n* = 8) or Gi-DREADDs (*n* = 8) in the anterior insula were water-deprived for 6 h during their light cycle (from 12 to 18 h) and given water during a 2-h session from the same drinking pipettes as used for the alcohol and sucrose drinking. After establishing stable baseline water consumption, rats were administered with CNO (10 mg/kg) 60 min before water access.

### Locomotor activity

Locomotor activity was measured using an open-field (43.2 × 43.2 × 30.5 cm) activity monitoring system equipped with infrared transmitters and receivers (ENV-515, Med Associates, St. Albans, VA, USA). Rats expressing the Gq-DREADDs in the anterior insula (*n* = 8) were first habituated to the arena during a 30-min session, and then tested in separate 30-min sessions after both saline and CNO injections (10 mg/kg) that were administered in a counterbalanced order 60 min before the sessions.

### Stereotaxic injection of viral vectors

Rats were anesthetized and maintained with isoflurane throughout the surgery (5% isoflurane induction concentration, 2.5% maintenance concentration, in oxygen, flow rate: 0.8–1 l/min). Animals were fixed in a stereotaxic frame (David Kopf Instruments, Tujunga, CA) with the incisor bar set to 3.3 mm below the interaural line. The coordinates for bilateral anterior insula injections were: anterior posterior (AP) +3.0 from bregma, mediolateral (ML) ± 4.2 from the sagittal suture, and ventrodorsal (VD) −6.1 from the skull surface, according to the Paxinos and Watson rat brain atlas^34^. The following vectors were used for DREADD and EGFP expression: the excitatory Gq-DREADD vector AAV8-hSyn1-hM3D(Gq)-mCherry [5.4 × 10^12^ vector genomes (vg)/ml], the inhibitory Gi-DREADD vector AAV8-hSyn1-hM4D(Gi)-mCherry (7.3 × 10^12^ vg/ml), and the EGFP control vector AAV8-hSyn1-EGFP (9.4 × 10^12^ vg/ml). The viral vectors were produced by the Viral Vector Facility (VVF) of the Neuroscience Center Zürich (Zentrum für Neurowissenschaften Zürich, ZNZ). The functionality of the DREADDs to alter alcohol drinking bidirectionally in the alcohol drinking assay was tested in a preliminary experiment, in which they were injected into the nucleus accumbens core. Each vector was injected with a 5-µl Neuros syringe (Hamilton) at a volume of 0.75 µl over 3 min, with an additional 3-min diffusion time. Carprofen (5 mg/kg s.c.; Norbrook Laboratories, Newry, UK) was administered for postoperative analgesia. After surgery, animals were returned to their home cages, and drinking sessions were resumed after two suspended sessions. In all experiments, DREADD expression was allowed to accumulate for 4 weeks before systemic CNO injections. In behavioral experiments, rats were habituated to the injection procedure by giving them saline injections prior to CNO. For mapping Gq-DREADD-induced brain activation with c-Fos expression, a previously described experimental design was used^35^. In brief, the AAV8-hSyn1-hM3D(Gq)-mCherry vector was injected unilaterally into the right anterior insula in alcohol-naïve AA rats (*n* = 9) as described, and challenged with CNO (10 mg/kg i.p.) after 4 weeks.

### Immunohistochemistry and c-Fos quantification

After completion of the experiments, animals were deeply anesthetized with a lidocaine–pentobarbital mix and perfused transcardially with phosphate-buffered saline (PBS, +4 °C, pH 7.4) followed by 4% paraformaldehyde (PFA, +4 °C, pH 7.4). The brains were removed and placed in PFA for 24-h post-fixation, after which PFA was replaced with 30% sucrose in PBS until brains were saturated (~4 days). Brains frozen in isopentane were stored at −80 °C until cutting with a freezing microtome into 40 µm coronal sections that were cryoprotected at −20 °C.

**Fig. 1:**
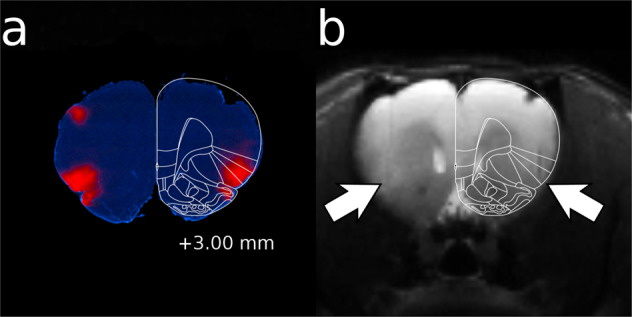
Gq-DREADD expression in the anterior insula verified by the extent of mCherry expression. Panel **a** shows a representative coronal brain section with the corresponding anatomical rat brain atlas section^34^ overlaid. The viral vector injections were aimed at the bregma level +3.0. Panel **b** shows the T2-weighted coronal image of the same subject. Arrows point to bilateral stereotaxic virus injection sites.

DREADD expression in the insula was visualized by immunohistochemical detection of mCherry tagged to DREADDs (see Supplementary Methods). Figure 1a shows a coronal section showing mCherry immunoreactivity at the Gq-DREADD injection site and its vicinity. The map indicated that in the anterior insula, DREADDs were expressed both in the agranular and dysgranular insular cortex, and to some extent in the gustatory cortex in the most posterior regions of the insula. Ventral to insula, some expression was seen in the piriform cortex, and dorsal to insula, in the primary somatosensory cortex, which most probably represents the leaked viral vector along the injection needle tract (for complete expression maps, see Supplementary Fig. S3). Noteworthy, viral vector injections did not cause structural damage or edema at the injection site, as indicated in Fig. 1b showing a T2-weighted image from a subject expressing Gq-DREADDs. Only subjects expressing either EGFP or mCherry in the anterior insula were included in the data analysis.

For assessment of c-Fos expression induced by CNO in rats with unilateral Gq-DREADDs in the insula, rats were sacrificed 90 min after CNO (10 mg/kg i.p.) injection. For quantification of nuclei positive for c-Fos, see Supplementary Methods. Nine brain areas were examined at three bregma coordinate levels: level +3.00 (anterior insula, orbitofrontal cortex, prelimbic cortex, infralimbic cortex), level +2.0 (nucleus accumbens core and shell), and level −2.5 (mediodorsal thalamic nucleus, ventromedial thalamic nucleus, central nucleus of the amygdala, basolateral amygdala, and posterior insula).

### Imaging protocol for phMRI

Sixteen AA rats expressing Gq-DREADDs and 12 EGFP-expressing (sham) rats, exposed to continuous 10% (v/v) ethanol in their home cages for 6 weeks and weighing around 350 g at the beginning of the imaging sessions, were used. The mean ethanol intake during the last ethanol access week was 8.1 ± 0.9 g/kg/body weight/day. For the MRI experiment, we adopted a minimally invasive protocol^36–40^ (see Supplementary Methods). After collection of T2-weighted anatomical references, image time series were acquired with a gradient echo sequence with a time resolution of 64 s per brain volume (see Supplementary Methods for details).

After acquisition of five reference images, a bolus of 30 mg Fe/kg of superparamagnetic iron oxide particles (USPIO Molday Ion, http://www.biopal.com/) was injected i.v. to sensitize the sequence to challenge-induced alterations in relative cerebral blood volume (rCBV)^41^. The use of exogeneous contrast agents provides dramatically increased sensitivity compared to conventional functional MRI methods^41^. Approximately 20 min (20 volumes) after the injection, necessary to reach the blood pool phase of the contrast agent, each subject received an i.v. challenge of saline vehicle (0.5 ml/kg). Thirty imaging volumes after the administration of vehicle, the animal received an i.v. injection of CNO (0.5 mg/kg in 0.5 ml). The total MRI time series consisted of 85 volumes (baseline, *n* = 5; contrast agent equilibration, *n* = 20; vehicle, *n* = 30; CNO, *n* = 30) for a duration of approximately 90 min.

### Statistical analysis

Sample sizes chosen for the behavioral and imaging experiments were determined on the basis of similar published studies. In all drinking experiments, the data were expressed as baseline drinking compared with the CNO injection day. The baseline was determined as the mean of the saline injection day and the following baseline day preceding the CNO injection. The differences between the groups expressing EGFP, Gq-DREADDs, or Gi-DREADDs were analyzed with two-way (session, DREADD vector) repeated measures ANOVA, with repeated measures on session. Differences between the baseline and CNO days were compared with paired *t*-tests. The c-Fos data were first analyzed with two-way ANOVA (hemisphere, brain area) with repeated measures on brain area, followed by comparison of the ipsi- and contralateral c-Fos expression with two-sample *t*-tests corrected for multiple comparisons (Benjamini–Hochberg procedure). In the repeated measures ANOVAs, assumption of sphericity was tested with Mauchly’s sphericity test. If sphericity was violated, the degrees of freedom of the *F*-distribution were corrected with the Greenhouse–Geisser procedure. For the phMRI data, rCBV time series were analyzed within the framework of the general linear model as previously described^36^. See Supplementary Methods for details. VOI-wise evaluation of rCBV alterations induced by CNO was conducted with a two-sample *t*-tests and corrected for multiple comparisons across volumes with FDR (*q* = 0.05). For all statistical analyses, *P* values from two-tailed tests were reported.

## Results

### Change in alcohol and sucrose drinking induced by insula DREADD activation

**Fig. 2:**
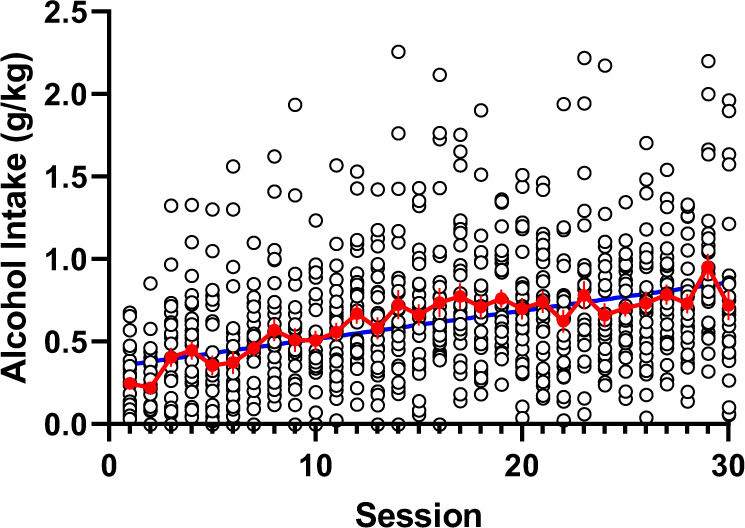
Acquisition on alcohol drinking by AA rats (*n* = 30) over 30 2-h sessions prior to stereotaxic viral vector injections. Shown are the alcohol intake (mean ± SEM), and all individual daily intakes with the linear regression line fitted.

During the acquisition of alcohol drinking in 2-h sessions every second day, AA rats gradually increased their alcohol consumption, shown by the significant deviation of the slope of linear regression from zero across the first 30 drinking sessions (*F*_1,898_ = 144.70, *P* < 0.001). The rats attained the mean intake of 0.80 ± 0.02 g/kg during the last three sessions and exhibited remarkable day-to-day and inter-individual variability in their drinking (Fig. 2). Using this model, we aimed at clarifying the involvement of the anterior insula in regulation of alcohol drinking. We injected both the excitatory and inhibitory DREADDs, as well as the control vector expressing EGFP into the anterior insula of AA rats. Four weeks after viral transfer of DREADDs into insula, we administered the rats with CNO 60 min prior to the session.

**Fig. 3:**
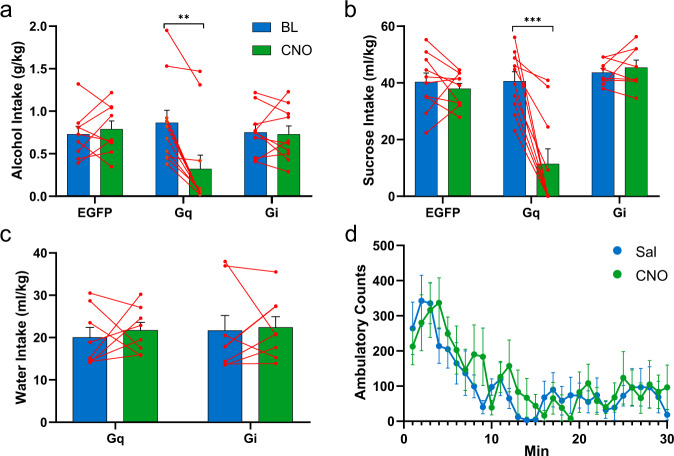
Anterior insula stimulation reduces the intake of liquid rewards. Effects of chemogenetic manipulation on alcohol (**a**), sucrose (**b**), and deprivation-induced water consumption (**c**), as well as locomotor activity (**d**). For alcohol, sucrose, and water drinking, the data are depicted as mean intake (±SEM) at the baseline (BL) and after CNO injections in groups expressing the EGFP control vector, the Gq-DREADDs, or the Gi-DREADDs. For alcohol drinking, data are derived from *n* = 9 EGFP-expressing, *n* = 11 Gq-DREADD-expressing, and *n* = 10 Gi-DREADD-expressing rats. For sucrose intake, data are from *n* = 10 EGFP-expressing, *n* = 10 Gq-DREADD-expressing, and *n* = 8 Gi-DREADD-expressing rats. For water intake, data represent *n* = 8 Gq-DREADD and *n* = 8 Gi-DREADD-expressing rats. Panel **c** depicts the effects of saline and CNO on locomotor activity (mean ambulatory counts ± SEM) in rats expressing Gq-DREADDs (*n* = 8) during a 30-min session. Asterisks indicate a significant difference of CNO-treated subjects from their baseline. **P < 0.01, ***P < 0.001, paired sample *t*-test.

Injection of CNO significantly altered alcohol drinking compared to the baseline, as revealed by a significant effect of session (*F*_1,2_ = 10.71, *P* = 0.003) and a significant session × vector interaction (*F*_2,27_ = 14.41, *P* < 0.001). In rats expressing the Gq-DREADDs, CNO decreased alcohol drinking significantly compared to the baseline (*t*_9_ = 4.00, *P* = 0.003) (Fig. 3a). Similarly, the sucrose-drinking rats exhibited both a significant effect of session (*F*_1,2_ = 20.10, *P* < 0.001) and session × vector interaction (*F*_2,25_ = 19.71, *P* < 0.001), produced by a significant attenuation of alcohol intake in the Gq-DREADD group (*t*_9_ = 5.68, *P* < 0.001) (Fig. 3b). Importantly, we found no significant differences between the experimental groups in alcohol and sucrose intake baselines preceding CNO injections (alcohol: *F*_2,29_ = 0.40, *P* = 0.68; sucrose: *F*_2,27_ = 0.36, *P* = 0.70).

Since stimulation of the anterior insula by Gq-DREADDs could have a general suppressive effect on behavior, we decided to test the effects of insula stimulation on physiologically motivated water drinking in rats following a 6-h water deprivation before the onset of the dark phase (Fig. 3c) and on locomotor activity in an open-field arena (Fig. 3d). In these experiments, CNO did not affect water drinking (session × vector interaction, *F*_1,14_ = 0.13, *P* = 0.72) nor locomotion (time × drug interaction, *F*_29,406_ = 0.79, *P* = 0.78).

### rCBV response induced by insula DREADD stimulation

**Fig. 4:**
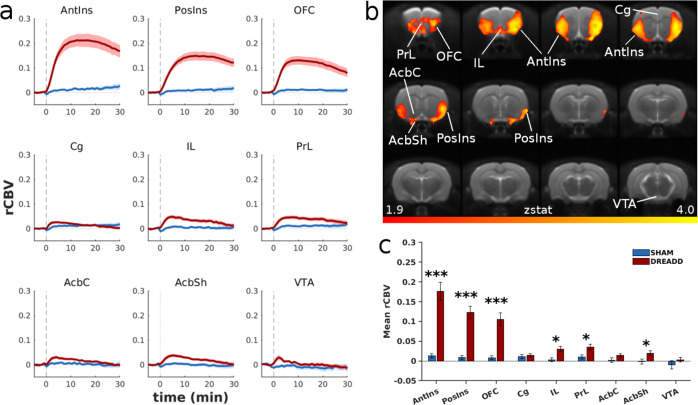
Brain activation induced by CNO (0.5 mg/kg i.v). Stimulation of Gq-DREADDs expressed in the anterior insula. **a** Time course of CNO-induced relative cerebral blood volume (rCBV) response (red line; *n* = 16) compared to the control group without DREADDs (blue line; *n* = 12). The dashed vertical line at *t* = 0 marks the time of CNO injection. **b** Anatomical localization of brain areas significantly activated by CNO in animals transfected with DREADDs versus controls (*z* > 1.9, *P* = 0.01). **c** Mean (±SEM) rCBV response in brain areas of interest after administration of CNO. ***P < 0.001, *P < 0.05, compared to the control group. AntIns anterior insular cortex, PosIns posterior insular cortex, OFC orbitofrontal cortex, Cg cingulate cortex, IL infralimbic cortex, PrL prelimbic cortex, AcbC nucleus accumbens core, AcbSh nucleus accumbens shell, VTA ventral tegmental area.

The effects of the anterior insula Gq-DREADD stimulation on alcohol and sucrose drinking could be mediated by a wider insula-centered circuitry. In order to gain unbiased information on the brain-wide activation pattern induced by bilateral insula stimulation, we expressed the Gq-DREADDs in the anterior insula and measured whole-brain activation with phMRI. Administration of CNO resulted in intense and sustained rCBV increase in the cortical rostrocaudal continuum comprising the anterior and posterior insula, as well as the orbitofrontal and medial prefrontal cortex, whereas there was no change in the same regions in subjects without DREADDs (Fig. 4a, b). Compared to the pre-CNO baseline, the anterior insula, posterior insula, and orbitofrontal cortex exhibited plateau levels of approximately 25%, 15%, and 10% above the baseline, respectively (Fig. 4a). Region-of-interest analysis of the magnitude of the rCBV signal change demonstrated significant differences in these areas compared to vehicle (both insular cortices: *P* < 0.001; orbitofrontal cortex: *P* < 0.001). We also detected a significant level of activation in the prelimbic and infralimbic cortex as well as the nucleus accumbens shell (*P* < 0.05) (Fig. 4c). When challenged with saline, no brain activation was observed either in the DREADD-expressing or the sham group (Supplementary Fig. S4).

### c-Fos expression following insula DREADD stimulation

**Fig. 5:**
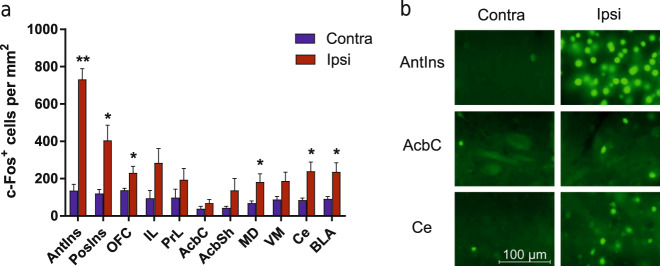
c-Fos expression induced by stimulation of unilaterally expressed Gq-DREADDs in the anterior insula. Presented in **a** are the mean (±SEM) (*n* = 9) densities of c-Fos immunoreactive nuclei in ten brain regions selected on the basis of anatomical connectivity with the anterior insula. Asterisks indicate a significant difference between the ipsilateral and contralateral brain regions *P < 0.05, **P < 0.01. Panel **b** shows examples of c-Fos expression in the contralateral and ipsilateral regions from a representative subject. AntIns anterior insula, PosIns posterior insula, OFC orbitofrontal cortex, IL infralimbic cortex, PrL prelimbic cortex, AcbC nucleus accumbens core, AcbSh nucleus accumbens shell, MD mediodorsal thalamus, VM ventromedial thalamus, Ce central nucleus of amygdala, BLA basolateral amygdala.

In order to corroborate the pattern of brain activation assessed by phMRI, we expressed Gq-DREADDs unilaterally in the anterior insula and quantified c-Fos expression in the ipsilateral and the unaffected contralateral brain regions following Gq-DREADD activation by CNO. In addition to brain areas revealed by phMRI, we also selected areas showing the anatomical connectivity of the anterior insula in previous tracing experiments^42,43^. Figure 5a, b shows that CNO induced widespread ipsilateral c-Fos expression in rats with the Gq-DREADDs expressed in the insula. Across the brain areas sampled, c-Fos expression in the ipsilateral hemisphere was significantly higher than in the contralateral one with no DREADD injections (*F*_1,16_ = 25.34, *P* < 0.001), and there were significant differences between the brain areas (*F*_4.6, 73.53_ = 14.89, *P* < 0.001; hemisphere × brain area interaction *F*_4.6, 73.53_ = 8.80, *P* < 0.001), with the anterior and posterior insula exhibiting the highest expression levels, followed by the orbitofrontal cortex, infralimbic cortex, mediodorsal thalamus, and amygdala.

## Discussion

The key finding of the present study is that anterior insula stimulation suppressed reward-related behavior, alcohol, and sucrose consumption, without influencing deprivation-induced water drinking or locomotor activity in a rat model of high alcohol preference. Using two independent but complementary methods, namely in vivo phMRI and postmortem c-Fos immunohistochemistry, we delineated a network of forebrain regions including the anterior and posterior insula, as well as the medial prefrontal cortex, mediodorsal thalamus, and amygdala associated with the behavioral effects. Notably, while identified in a data-driven manner, all structures have well-established functions in the control of appetitive behaviors. Our results may have translational relevance given that the insula is emerging as a target for interference in addictive as well as eating disorders^44,45^.

Our data do not directly point to any specific insula function that the Gq-DREADD activation could have altered in a manner conducive for alcohol and sucrose intake suppression. Because the insula mediates taste processing^46^ and the interoceptive effects of alcohol^47^, changes in these insula functions could be reflected in the amount of liquid rewards consumed. However, human imaging literature suggests that the insula is not only involved in the evaluation of subjective outcomes but also in the anticipation of them^48^. This was demonstrated experimentally by electrical microstimulation of the anterior insula during the presentation of the conditioned stimulus associated with a juice reward in rhesus monkeys^49^. During this period, insula stimulation reduced appetitive approach behavior. In another study conducted in monkeys, electrical stimulation of specific regions of the anterior agranular/dysgranular insula elicited disgust-like responses in subjects presented with their preferred food, without evoking unpleasant or painful sensations^50^. Similarly, we found that chemogenetic anterior insula stimulation failed to affect normal ambulatory activity or water drinking motivated by mild deprivation, in line with previously reported selective attenuation of high fat binge intake, but not food-deprivation-induced feeding^51^. Together, these data suggest that anterior insula stimulation may attenuate hedonic, rather than homeostatic, consumption. It remains to be tested whether the specific effects on alcohol and sucrose drinking can be found in heterogeneous rodent strains that do not exhibit high alcohol preference or in female rats.

The lack of effects by chemogenetic silencing of the insula may seem perplexing in the light of previous lesion and inactivation studies in addiction models. However, it has been shown that Gi-DREADD activation produces only a partial suppression of neuronal firing in vivo, an effect remarkably smaller than that of Gq-DREADD-induced stimulation^52,53^. Chemogenetic inhibition may thus rather dampen than eliminate neural activity, and lead to less drastic behavioral changes than lesions or inactivation. It is also possible that the effects of anterior insula inhibition may depend on the behavioral context, as suggested by data that insula silencing is effective in situations where alcohol seeking or consumption is associated with adverse consequences. For example, optogenetic inhibition of the projection form the insula to the nucleus accumbens suppressed only quinine-adulterated, but not quinine-free alcohol drinking^54^, and anterior insula inactivation attenuated alcohol seeking in the punishment context^55^. Recent data suggest that chemogenetic insula inhibition may even increase alcohol self-administration^56^.

phMRI revealed that stimulation of the anterior insula Gq-DREADDs by CNO induced significant neural activation along a cortical band extending rostrally to the prefrontal cortex and caudally to the posterior insula and entorhinal cortex from the site of DREADD expression. This finding is in agreement with the propagation of anterior insula excitation by repeated electrical stimulation along the rostrocaudal axis, parallel to the rhinal sulcus^57^. The activation pattern is also consistent with the anatomical connectivity of the anterior insula with the medial prefrontal cortex (prelimbic and infralimbic cortex), lateral orbital cortex, and posterior insula^43,58^, and is thus largely mediated by local cortical circuits. In addition to cortical activation, a more detailed c-Fos mapping indicated activation in the mediodorsal thalamic nucleus and the amygdaloid nuclei, which can be predicted by the reciprocal anatomical connections of the anterior insula with these brain areas^42,43^. Activation of several downstream brain areas following insula stimulation suggest that these areas, including prefrontal and orbitofrontal cortices, as well amygdala, could belong to the extended functional circuit involved in the attenuation of appetitive behavior. For example, activation of the insula projection to the central amygdala could induce avoidance behavior^23,59^. However, our data do not provide direct evidence that these functionally connected networks contributed to the behavioral changes. It is also possible that stimulation or silencing of specific insula projections could produce behavioral effects that are different from targeting insula somas. Because we used an efficient neuron-specific human synapsin 1 (hSyn) promotor for driving DREADD expression, also cortical inhibitory interneurons could have been activated, dampening ensembles of insula projection neurons. On the other hand, with high AAV vector titers comparable to the ones used here, the hSyn promotor was shown to target predominantly excitatory neurons^60^.

Although phMRI measurements were performed under light anesthesia, previous work indicates that phMRI responses are preserved under sedation conditions similar to those used in our study^32,36,40^. In addition, the phMRI activation maps were in good agreement with the c-Fos mapped activation and reveal the neural origin of the observed activation. These data also suggest that anesthesia had no major effects on neuronal stimulation produced by CNO Gq-DREADD activation. The slightly different activation patterns revealed by phMRI and c-Fos mapping could reflect the fact that phMRI is based on hemodynamic changes, whereas c-Fos serves as an indirect molecular activity marker. In addition, rodents have been shown to exhibit lateralized brain activation during aversive conditioning, e.g., increased right amygdala immediate-early gene expression^61,62^. It is therefore possible that CNO injection procedures to activate right insula DREADDs could have contributed to ipsilateral amygdala c-Fos expression. Methodologically, the combination of these three methods, site-specific DREADD-mediated brain stimulation, phMRI, and c-Fos mapping comprised a powerful tool for network analysis and identified insula connections for further functional testing.

Our experiments were conducted in a translational framework that aimed at guiding the use of neuromodulatory approaches such as repetitive TMS (rTMS) for the treatment of alcohol use disorders. Instead of using TMS, focal electrical, or optogenetic stimulation, we used non-invasive chemogenetic neural manipulation to alter insula neural activity. The difficulty of delivering high-intensity TMS with good spatial resolution limits the experimental use of TMS in rodents^63^. An additional challenge with electrical or optogenetic stimulation is their integration with in vivo imaging in the MRI setting.

Translation of DREADD-induced neural activation to rTMS parameters is difficult, because these techniques alter neuronal functioning in a fundamentally different manner. In addition, although chemogenetic neural control has an excellent spatial resolution, its temporal resolution is poor compared to rTMS. rTMS is delivered as trains of pulses over several minutes, whereas systemic CNO administration induces a sustained increase in CNO plasma concentration, enhancing neuronal activity through Gq-DREADDs for several hours^64^. However, it could be argued that ultimately changes in brain activation produced both by DREADDs and rTMS may lead to plasticity at the synaptic level. For example, Gq-DREADD activation increased synaptic transmission and long-term potentiation in hippocampal glutamatergic neurons^65^, and similarly cortical excitability induced by high frequency rTMS was hypothesized to emulate aspects of activity-dependent plasticity^66,67^. Therefore, the effects of chemogenetic manipulation and rTMS on neuronal excitability may converge at the facilitation of synaptic connections.

Translational intervention based on neural circuits was successfully demonstrated recently in cocaine addiction. Optogenetic stimulation of the prelimbic cortex attenuated cocaine seeking in rats^68^, prompting the use of high-frequency rTMS targeting the functional human homolog of prelimbic cortex, the dorsolateral prefrontal cortex. In cocaine-addicted patients, rTMS reduced cocaine intake and craving^69^, as well as improved inhibitory control and attention^70^. There have been relatively few studies on insula-targeted neuromodulation in substance use disorders. Interestingly, rTMS of the bilateral lateral prefrontal cortex and insula applied following presentation of smoking cues attenuated cigarette consumption^45^, which agrees with the suppression of alcohol drinking following insula stimulation with Gq-DREADDs. In contrast, rTMS targeting bilateral insula but excluding anterior prefrontal areas failed to decrease alcohol craving or drinking compared to sham stimulation, which may indicate that concomitant prefrontal cortex stimulation is needed for clinical efficacy^71^. Characterization of the neural elements underlying the observed effects or lack thereof is crucial for further development of neuromodulatory interventions in substance use disorders.

In summary, we showed that insula stimulation by Gq-DREADDs significantly reduced the intake of liquid rewards, alcohol and sucrose, and induced intense brain activation in both the anterior and posterior insula, areas of the prefrontal cortex, as well as thalamic and amygdaloid nuclei that have reciprocal connections with the insula. These data indicate that the insula is an important cortical hub for mediating reward-related behaviors and could be a target for circuit-based therapy. Future work is needed to elucidate the exact processes affected by insula stimulation and the specific neuron types and circuits involved.

## Supplementary information

Supplemental material
