# Supplementary material for: Anterior insula stimulation suppresses appetitive behavior while inducing forebrain activation in alcohol-preferring rats
Source: Transl Psychiatry. 2020 May 18;10:150. doi: 10.1038/s41398-020-0833-7 (PMC7235223; doi:10.1038/s41398-020-0833-7)
Supplement: Supplementary file 1 — Supplemental material [file 41398_2020_833_MOESM1_ESM.docx]

**Supplementary Materials**

**Supplementary methods**

*Immunohistochemistry and c-Fos quantification*

After completion of the experiments, animals were deeply anesthetized with a lidocaine-pentobarbital mix and perfused transcardially with phosphate-buffered saline (PBS, +4 °C, pH 7.4) followed by 4% paraformaldehyde (PFA, +4 °C, pH 7.4). The brains were removed and placed in PFA for 24-h post-fixation, after which PFA was replaced with 30% sucrose in PBS until brains were saturated (~4 days). Brains frozen in isopentane were stored at -80 °C until cutting with a freezing microtome into 40 µm coronal sections that were cryoprotected at -20°C.

DREADD expression in the insula was visualized by immunohistochemical detection of mCherry tagged to DREADDs. Brain sections were washed three times in PBS for 5 minutes and blocked at room temperature for 1 h in blocking solution containing 3% BSA, 10% donkey serum, and 0.3% Triton solubilized in PBS. Sections were incubated overnight at +4°C with the rabbit anti-mCherry primary antibody (ab167453, Abcam), followed by a 2 h incubation with the secondary donkey anti-rabbit antibody (ab150076, Abcam) at RT. Sections were then mounted on microscopic slides, coverslipped with Vectashield-DAPI, and imaged with a Zeiss AxioImager.Z1 upright epifluorescent microscope using the ZEN Blue software.

For quantification of c-Fos expression induced by CNO in rats with unilateral Gq-DREADDs in the insula, rats were sacrificed 90 min after CNO (10 mg/kg i.p.) injection. After extracting and cutting the brains (see above), 40-µm sections were washed three times for 5 minutes in PBS, placed in trisodium citrate solution at 80°C for 15 minutes (10 mM, pH 7.4, in PBS), and allowed to cool down to room temperature. After washing in PBS, sections were incubated in blocking buffer (1-3% bovine serum albumin, 10% goat serum, and 0.3% triton x-100 dissolved in PBS) for 1 h. Sections were then incubated in mouse anti-c-Fos (ab208942, Abcam, 1:500) at +4°C overnight, washed, and visualized with a goat anti-mouse fluorescent secondary antibody (Alexa Fluor 488, ab150113, Abcam, 1:800) for 2 h in room temperature. The sections were mounted on microscope slides and coverslipped with Vectashield-DAPI. Sections were examined first on a Zeiss AxioImager.Z1 upright epifluorescence microscope at 5x magnification with both a DAPI and mRFP filter to verify the unilateral expression of the mCherry-tagged DREADD. For c-Fos quantification, brain sections were imaged under a 40x magnification with the GFP filter. Nuclei positive for c-Fos were counted from three replicate sections bilaterally for each brain area using an ImageJ macro, averaged over the replicates, and expressed as c-Fos positive cells per mm^2^. Eleven brain areas were examined at three bregma coordinate levels: level +3.00 (anterior insula, orbitofrontal cortex, prelimbic cortex, infralimbic cortex), level +2.0 (nucleus accumbens core and shell), and level -2.5 (mediodorsal thalamic nucleus, ventromedial thalamic nucleus, central nucleus of the amygdala, basolateral amygdala, and posterior insula).

*Animal preparation for phMRI*

Animals were initially anesthetized with 3% isoflurane (Vetflurane, VIRBAC SA, Carros, France) in O2:N2O (3:7), which was then reduced to 1.5% for maintenance during preparation (1). The tail vein was cannulated with a 26G intravenous catheter to allow the injection of contrast agent (30 mg Fe/kg of superparamagnetic iron oxide particles) (USPIO Molday Ion, http://www.biopal.com/). Subsequently, a bolus of 0.05 mg/kg/ml of medetomidine (Domitor, 1 mg/ml, Orion Corporation, Espoo, Finland) was injected intraperitoneally, followed 15 minutes later by a continuous infusion of medetomidine diluted 1:5 in saline at a rate of 1 ml/kg/h through a catheter inserted subcutaneously (1-5). After that isoflurane was tapered to 0.3%. During the scan session, respiration rate, heart rate, and blood oxygen saturation were monitored with a pulsoximeter (https://www.starrlifesciences.com). CO_2_ concentration in the blood was also assessed using a TCM4 Transcutaneous Blood Gas Analyzer (https://www.radiometer.com). Rectal temperature was maintained at 37°C during the experiments by a feedback-controlled, water-circulating heating pad. We acquired a low resolution scout image and a T2-weighted anatomical images using a turbo spin-echo sequence (RARE, TR = 3500 ms, TE = 48 ms, RARE factor = 8, FOV = 35×35×24 mm, 256×256 matrix, 24 contiguous 1 mm slices), followed by a gradient echo sequence with a time resolution of 64 s per brain volume (Segmented FLASH, TReff = 200 ms, TE = 5 ms, NEX = 4, FOV = 35×35×16 mm, 100×100 matrix, 16 contiguous 1 mm slices). At the end of the fMRI experiment, medetomidine anaesthesia was terminated by an intraperitoneal injection of atipamezole (Antisedan, 0.1 mg/kg; Orion Corporation, Espoo, Finland).

*phMRI data analysis*

MRI images were processed with MATLAB (https://www.mathworks.com) and FSL (http://fmrib.ox.ac.uk/fsl). Raw data were converted to NIFTI format and the voxel size was scaled by a factor of 10 for compliance with FSL algorithms. The minimal preprocessing pipeline included motion correction and slice timing correction; pharmacological time series were then co-registered by 12 DOF affine transformations to structural T2w anatomical references and resampled to a rat brain template in standard space (6). After rigid body transformation, nonlinear warping to standard space was applied to further minimize geometric distortions. Signal intensity changes were converted into relative CBV using a constrained exponential model of the gradual elimination of the contrast agent from the blood (7). The rCBV time series were calculated covering 15 volumes as baseline and 60 volumes post-challenge (vehicle and CNO) window. FWHM spatial smoothing and two boxcar regressors (vehicle: off (1:15), on (16:76), CNO: off (1:45), on (46:76)) were used to capture the signal change induced by vehicle or CNO (6). To avoid spurious contribution of the CNO challenge in the calculation of vehicle parameter estimates, the two regressors were orthogonalized. Higher level group inference was carried out using the FSL-FEAT software (FLAME 1); z-statistics images were thresholded using clusters determined by z > 1.9 and a corrected cluster significance of p = 0.01. An additional volume of interest (VOI) -wise analysis of phMRI time courses for vehicle or CNO challenges was conducted by extracting from preprocessed phMRI datasets 62 bilateral volumes of interest using a digital reconstruction of the rat brain atlas (8), co-registered with the MRI template (6). For each VOI, the mean rCBV value over a 30-min time window (1–30 min post injection) covering the response produced by CNO was used for statistical comparison. A similar approach has been recently applied under similar experimental conditions (9).

*Pharmacokinetic measurements*

Plasma samples (150 µl) were collected from 8 naive Wistar rats at different time points (15, 30, and 60 min for the IV dose and 30, 60, 90, and 120 min for the IP administration). Animals were anesthetized with isoflurane (3%) and an IV catheter was implanted to allow for intravenous administration of CNO and collection of blood samples under isoflurane anaesthesia (2%).

Plasma samples were centrifuged for 20 min. A 50 µl aliquot was then transferred into 96-deepwell plate and added with 150 µl of the extraction solution, consisting of cold acetonitrile spiked with clozapine-d4 as internal standard. After agitation (3 minutes) the plate was centrifuged at 3000 g for 20 minutes at 4°C. 80 µl of supernatant were then transferred in a 96-well plate and added with 80 µl of water. Reference standards of clozapine-N-oxide, clozapine and N-desmethyl-clozapine were spiked in naïve rat plasma to prepare a calibration curve over the 1 nM – 10 µM range. Three quality controls samples were also prepared by spiking the compounds in blank rat plasma to final 20, 200 and 2000 nM concentrations. Calibrators and QCs were extracted with the same extraction solution used for the plasma samples. Plasma levels of clozapine-N-oxide, clozapine and N-desmethyl-clozapine were monitored on a ACQUITY UPLC/MS TQD system consisting of a TQD (Triple Quadrupole Detector) mass spectrometer equipped with an electrospray ionization interface; 3ul of each sample were injected on a reversed phase column (Acquity UPLC HSS T3 2.1 x 50 mm, 1.8 µm particle size) and separated with a gradient of acetonitrile (10% to 50% B in two minutes). Column and UPLC-MS system were purchased from Waters Inc. Milford, USA. Flow rate was set 0.5 ml/min. Eluents were A = water and B = acetonitrile, both added with 0.1% formic acid. Analysis was performed in ESI+ ionization mode. Compounds were quantified by monitoring their MRM peak areas.

**References**

1. Gozzi A, Schwarz A, Crestan V, Bifone A. Drug–anaesthetic interaction in phMRI: the case of the psychotomimetic agent phencyclidine. Magn Reson Imaging. 2008;26:999-1006.

2. D'Souza DV, et al. Preserved modular network organization in the sedated rat brain. PloS one. 2014;9:e106156.

3. Pawela CP, et al. A protocol for use of medetomidine anesthesia in rats for extended studies using task-induced BOLD contrast and resting-state functional connectivity. Neuroimage. 2009;46:1137-47.

4. Tambalo S, et al. Functional Magnetic Resonance Imaging of Rats with Experimental Autoimmune Encephalomyelitis Reveals Brain Cortex Remodeling. J Neurosci. 2015;35:10088-100.

5. Paasonen J, Stenroos P, Salo RA, Kiviniemi V, Gröhn O. Functional connectivity under six anesthesia protocols and the awake condition in rat brain. Neuroimage. 2018;172:9-20.

6. Schwarz AJ, et al. A stereotaxic MRI template set for the rat brain with tissue class distribution maps and co-registered anatomical atlas: application to pharmacological MRI. Neuroimage. 2006;32:538-50.

7. Schwarz AJ, Reese T, Gozzi A, Bifone A. Functional MRI using intravascular contrast agents: detrending of the relative cerebrovascular (rCBV) time course. Magn Reson Imaging. 2003;21:1191-200.

8. Paxinos G, Watson C. The Rat Brain in Stereotaxic Coordinates, 6th ed. Academic Press, London, 2007.

9. Gozzi A, et al. Neuroimaging Evidence of Altered Fronto-Cortical and Striatal Function after Prolonged Cocaine Self-Administration in the Rat. Neuropsychopharmacology. 2011;36:2431-40.

**Supplementary figure legends**

**Figure S1**. Brain activation induced by CNO in naïve rats. Time course of challenge-induced relative cerebral blood volume (rCBV) response (green line; n = 10). The dashed vertical line at t = 0 marks the time of CNO injection. AntIns: anterior insular cortex; PosIns: posterior insular cortex; OFC: orbitofrontal cortex; Cg: cingulate cortex; IL: infralimbic cortex; PrL: prelimbic cortex; AcbC: nucleus accumbens core; AcbSh: nucleus accumbens shell; VTA: ventral tegmental area.

**Figure S2**. Pharmacokinetic quantification of CNO and clozapine concentration in plasma as a function of time. a) Intraperitoneal (IP) administration of 10 mg/kg CNO. b) Intravenous injection of 0.5 mg/kg CNO. These doses and routes of administration were employed in this study for behavioral and phMRI experiments, respectively.

**Figure S3**. Localization of DREADDs in the brain verified by the extent EGFP and mCherry (Gq- and Gi-DREADDs) expression at three different bregma levels in subjects from (A) the alcohol drinking experiment and (B) the rCBF experiment. The viral vector injections were aimed at the bregma level +3.0. The red color shows the maximum extent of expression and the blue color the minimum extent. In Panel C, anterior insula is indicated by red color, and the intended location of injection cannula tips by an asterisk.

**Figure S4**. Brain activation induced by saline (i.v.) in rats expressing Gq-DREADDs in the anterior insula. a) Time course of saline-induced relative cerebral blood volume (rCBV) response (red line; n = 16) compared to the sham group without DREADDs (blue line; n = 12). The dashed vertical line at t = 0 marks the time of saline injection. b) Anatomical localization of brain areas activated by saline in animals transfected with DREADDs versus controls (z > 1.9, p = 0.01). c) Mean (± SEM) rCBV response in brain areas of interest after administration of saline. AntIns: anterior insular cortex; PosIns: posterior insular cortex; OFC: orbitofrontal cortex; Cg: cingulate cortex; IL: infralimbic cortex; PrL: prelimbic cortex; AcbC: nucleus accumbens core; AcbSh: nucleus accumbens shell; VTA: ventral tegmental area

**Supplemental figures**


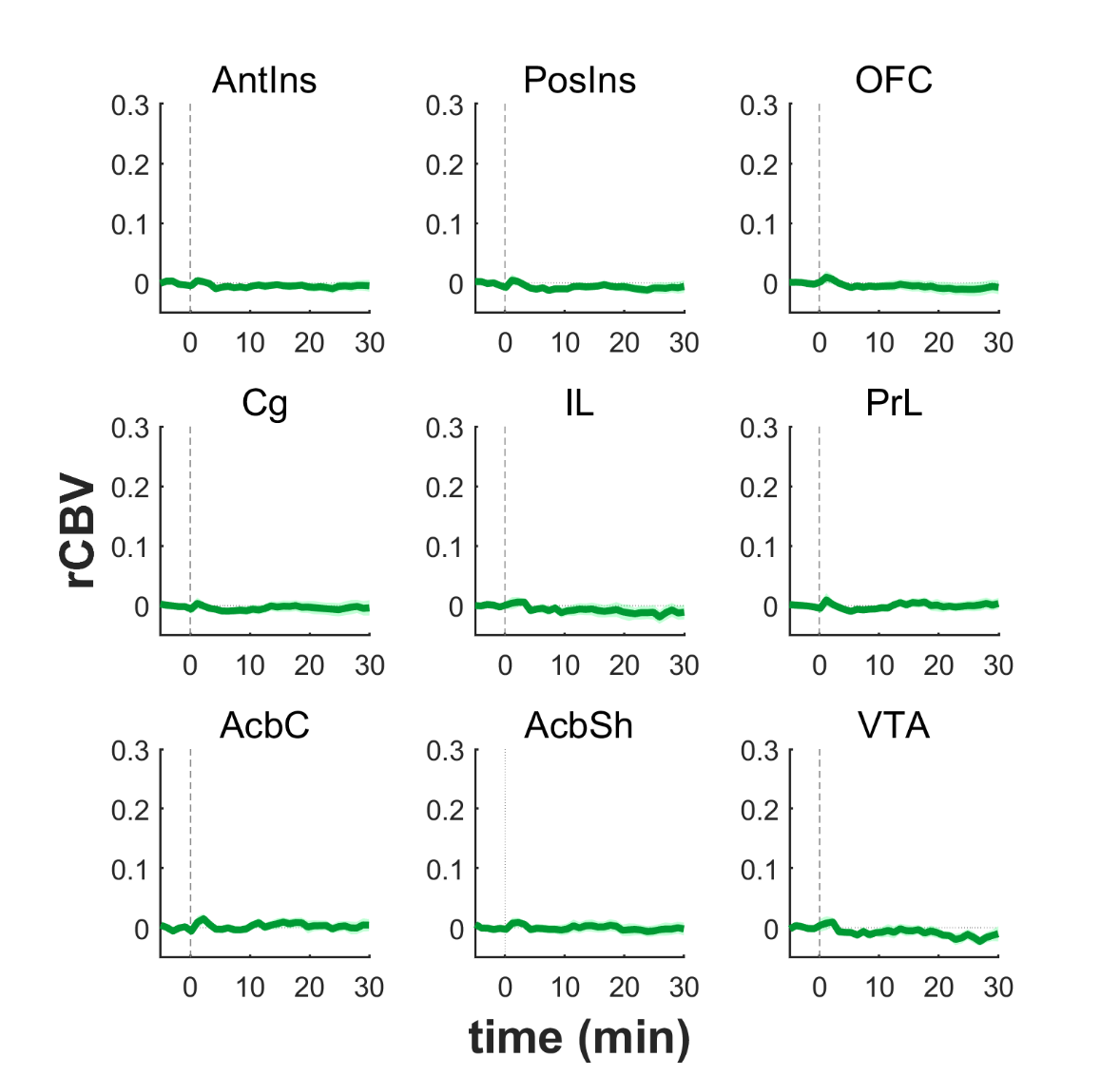


**Figure S1**


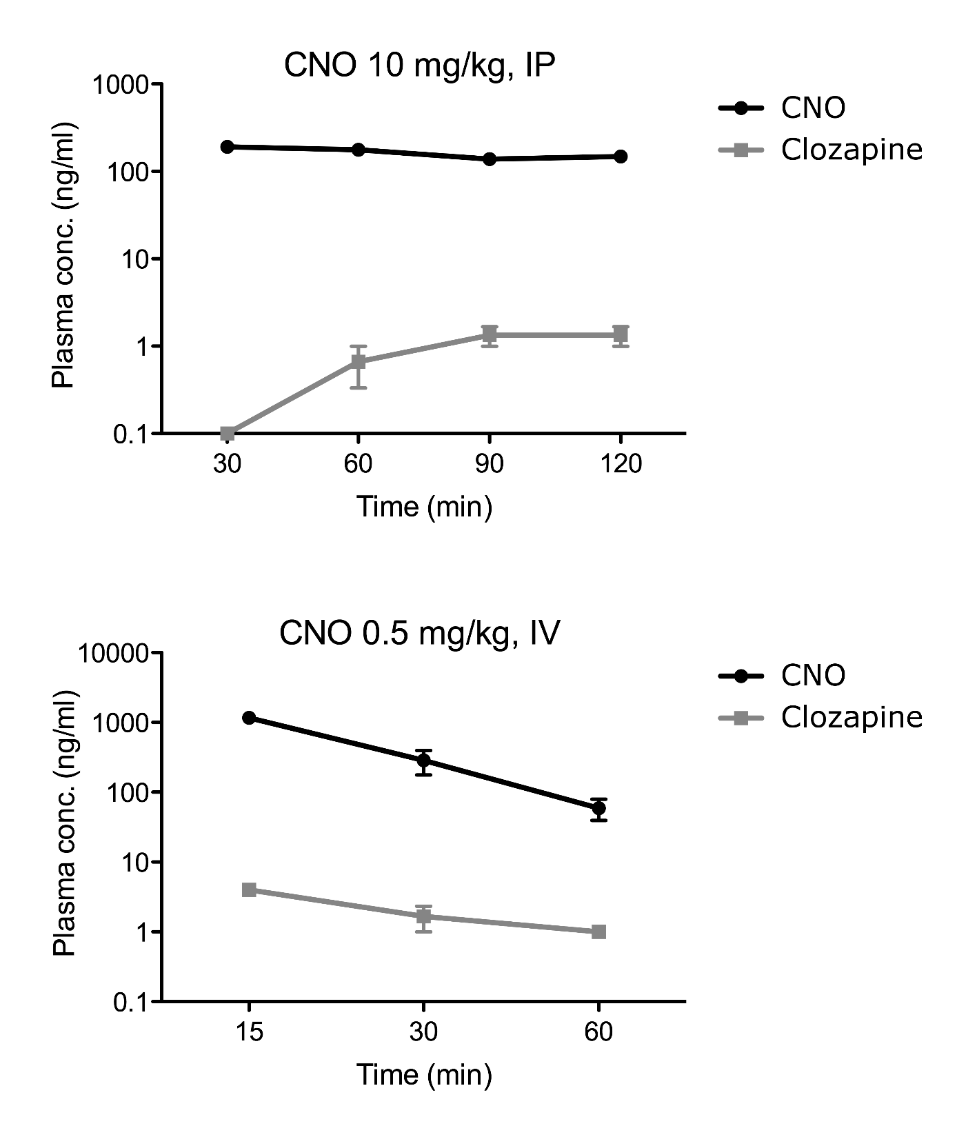
**Figure S2**

**Figure S3**


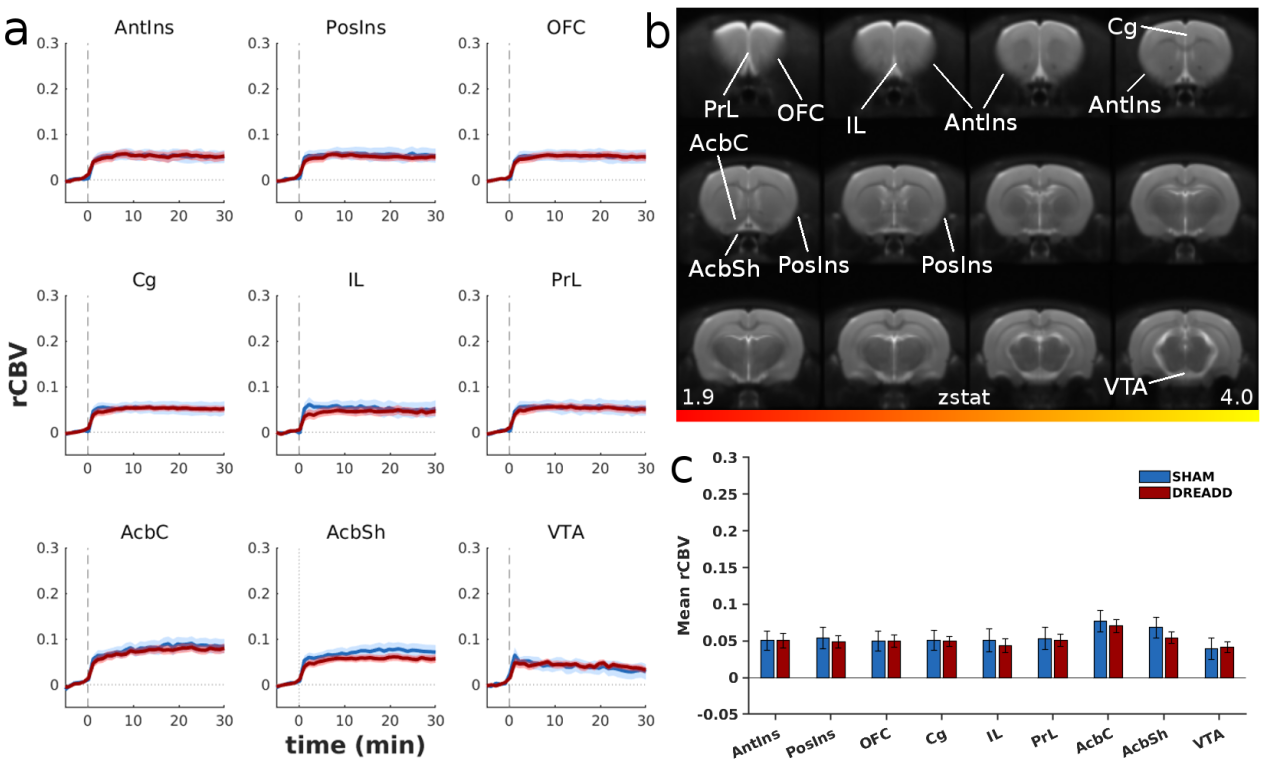


**Figure S4**
